# Anoikis-related genes combined with single cell sequencing: Insights into model specification of lung adenocarcinoma and applicability for prognosis and therapy

**DOI:** 10.3389/fcell.2023.1125782

**Published:** 2023-04-24

**Authors:** Yiyi Zhou, Zhenli Hu

**Affiliations:** Department of Respiratory and Critical Care Medicine, Changhai Hospital, Shanghai, China

**Keywords:** lung adenocarcinoma, anoikis, cancer prognosis, ScRNA-seq, immune microenvironment

## Abstract

**Background:** Anoikis has therapeutic potential against different malignancies including lung adenocarcinoma. This study used anoikis and bioinformatics to construct a prognostic model for lung adenocarcinoma and explore new therapeutic strategies.

**Methods:** Several bioinformatic algorithms (co-expression analysis, univariate Cox analysis, multivariate Cox analysis, and cross-validation) were used to screen anoikis-related genes (ARGs) to construct a risk model. Lung adenocarcinoma patients were divided into training and testing groups at a ratio of 1:1. The prognostic model was validated by risk score comparison between high- and low-risk groups using receiver operating characteristic curve (ROC), nomograms, independent prognostic analysis and principal component analysis. In addition, two anoikis-related genes patterns were classified utilizing consensus clustering method and were compared with each other in survival time, immune microenvironment, and regulation in pathway. Single cell sequencing was applied to analyze anoikis-related genes constructed the model.

**Results:** This study demonstrated the feasibility of the model based on seven anoikis-related genes, as well as identifying axitinib, nibtinib and sorafenib as potential therapeutic strategies for LUAD. Risk score based on this model had could be used as an independent prognostic factor for lung adenocarcinoma (HR > 1; *p* < 0.001) and had the highest accuracy to predict survival compared with the clinical characteristics. Single cell sequencing analysis discovered Keratin 14 (KRT14, one of the seven anoikis-related genes) was mainly expressed in malignant cells in various cancers.

**Conclusion:** We identified seven anoikis-related genes and constructed an accurate risk model based on bioinformatics analysis that can be used for prognostic prediction and for the design of therapeutic strategies in clinical practice.

## Introduction

Lung cancer is one of the malignancies with the highest morbidity and mortality (Siegel et al., 2021). In 2022, an estimated 236,740 new cases and an estimated 130,180 deaths are covered by lung carcinoma. On account of the hysteretic nature of datum, the 5-year relative survival is accessible lags 4 years behind the current year, with 22.9% from 2012 to 2018 (Cancer Stat Facts: lung and bronchus cancer, 2022). The most common subtype of lung cancer including never-smokers is lung adenocarcinoma (LUAD) (Lee et al., 2019; Devarakonda et al., 2021). Research advances have improved the early diagnosis of patients, thus leading to a better prognosis. Several treatment options are available including immunotherapy, targeted therapy, chemotherapy, radiotherapy, and surgical management (pneumonectomy and lung transplantation) (Glanville and Wilson, 2018; Wang et al., 2022a; Gross et al., 2022; Huo et al., 2022; Nguyen et al., 2022). However, LUAD is characterized by considerable heterogeneity in clinical features, pathological characteristics, and molecular alterations, as well as genomic instability (Geng et al., 2021; Okudela et al., 2021). The prevalence of lung tumors in non-smokers has increased recently, despite the known association between smoking and lung cancer (Jeon et al., 2018), underscoring the need to elucidate the underlying molecular mechanisms and develop effective therapies.

Recently, programmed cell death has been the focus of research aimed at developing new cancer treatment strategies. There are several cell death mechanisms including apoptosis (Green, 2022), autophagy (Russell and Guan, 2022), cuproptosis (Tsvetkov et al., 2022), ferroptosis, pyroptosis and necroptosis (Tang et al., 2020). A key factor in maintaining cell homeostasis is adhesion of cells to matrix, and disruption of this interaction can adversely affect cell survival (Chiarugi and Giannoni, 2008). Disengagement between cells and matrix can lead to long-time cells suspension and cell death. This cell death form has been termed “anoikis” (Frisch and Francis, 1994). The presence of anoikis may hold back the reattachment of detached cells and malformation of cells development. Anoikis after the loss of cell anchorage is associated with cells development, tissue homeostasis and malignancies (Danial and Korsmeyer, 2004; Chiarugi and Giannoni, 2008; Simpson et al., 2008). After escaping from the adhesion of extracellular matrix and intercellular contact, tumor cells survived by paracrine and paracrine mechanisms against apoptosis, and regained the ability to attach to spread, metastasize and invade. Anti-anoikis is an important feature of tumor metastasis, which enables tumor cells to spread to distant organs through the circulatory system (Simpson et al., 2008; Chaffer et al., 2016). The regulation of anoikis in lung carcinoma mainly includes extracellular matrix (ECM) and cell adhesion, cell detachment and directional migration, signal transduction and regulators (Wang et al., 2022b). Anchor independent survival of tumor cells requires detachment from ECM matrix (Pickup et al., 2014; Enkhbat et al., 2022). The inhibition of the overexpression of fibronectin in cell aggregation during detachment has been proved that can enhance anoikis in cancer (Han et al., 2021). In the drug resistance experiments of lung adenocarcinoma, upregulated Laminin 5 can reduce anoikis through activating integrin/focal adhesion kinase (FAK) signaling (Kodama et al., 2005). Generally, cells following the loss of adhesion won’t express epidermal growth factor receptor (EGFR) and then induce apoptosis. Research indicated that family with sequence similarity 188 member B (FAM188B) can prevent the degradation of EGFR and lead to the re-adhesion of lung cancer cells to the ECM (Jang et al., 2021). Non-coding RNA and IL-13 receptor subunit alpha-2 (IL13Rα2) which both involved in PI3K/AKT pathway regulation inhibit anoikis and result in lung carcinoma metastasis and grave prognosis (Xie et al., 2015; Tian et al., 2020). Anoikis play an important role in LUAD; however, the potential role of ARGs in LUAD still needs to be examined to date. Here, we hypothesized that ARGs play a role in the occurrence and development of LUAD, and constructed models to explore the mechanism of ARGs in LUAD.

The study identified ARGs and constructed a prognostic model based on the ARGs signature, which revealed the association of these markers with prognostic prediction and treatment modalities in LUAD, as well as their role in immune microenvironment, and drug sensitivity. Additionally, single cell data were used to analyze these genes for constructing the model. The functions of ARGs in the classification of LUAD were evaluated based on the TCGA and GEO datasets.

## Materials and methods

### Data acquisition

Transcriptome profiling (RNAseq) data and relevant clinicopathological information in LUAD, were obtained from The Cancer Genome Atlas (TCGA) database (https://portal.gdc.cancer.gov/) and Gene Expression Synthesis Database (GSE41271, GEO, https://www.ncbi.nlm.nih.gov/geo). ARGs were incorporated through a systematic search and processing from the Human Gene Database (GeneCards, https://www.genecards.org/) and Harmonizome platform (https://maayanlab.cloud/Harmonizome/). The scRNA-seq database from Tumor Immune Single-cell Hub 2 (TISCH2, http://tisch.comp-genomics.org/) website was analyzed to explore the model-related genes expression in pan-cancer.

### Construction of a prognostic network and CNV analysis

Univariate Cox regression analysis was performed using R software to screen differentially expressed genes (DEGs). We used “igraph,” “psych,” “reshape2” and “RColorBrewer” R packages to examine the correlation between ARGs and risk factors. After downloading copy number variation (CNV) data from UCSC Xena database (http://xena.ucsc.edu/), we processed the CNV data to calculate frequency of mutation using the Perl script algorithm. The “RCircos” package was used for graphical representation.

### Consensus clustering analysis and principal component analysis of ARGs

According to the consensus level of ARGs, the cohort from TCGA and GEO was allocated into several clusters utilizing the R package “ConsensusClusterPlus”. The fitness of the classification was judged based on the principal component analysis (PCA) algorithms. “survival” and “survminer” were also applied to construct curve plotting and compare the survival between different clusters. The other graphical output consisted of heatmap and boxplots using “pheatmap” and “boxplots”.

### Pathway enrichment analysis of ARGs

Gene set variation analysis (GSVA), an extension of gene set enrichment (GSE) method, are performed for unsupervised classification of a sample based on the variation of pathway activity (Hanzelmann et al., 2013). Gene set enrichment analysis (GSEA) is an analytical method and focused on gene sets which share common biological function, enrichment pathways and chromosomal location (Subramanian et al., 2005; Cao et al., 2019). The Kyoto Encyclopedia of Genes and Genomes (KEGG) analyses were performed to present the discrepancy of pathways between those ARGs clusters. The process was completed by the “GSEABase,” “GSVA” and “clusterProfiler” R package with the screening criteria adjusted *p* < 0.05.

### Establishment and validation of an anoikis-related risk model

To identify prognostic ARGs, univariate Cox regression analysis was performed using R software. LUAD patients were randomly divided into training and testing cohorts at a ratio of 1:1. The “glmnet” R package was used to perform the least absolute shrinkage and selection operator (LASSO) regression to analyze prognostic candidates. With the optimization criterion of least error, cross validation was performed to screen target ARGs, which were applied to the optimal multivariate Cox model. Subsequently, an ARGs prognostic signature was constructed utilizing the “survival” package based on the coefficient and hazard ratio (HR). The risk score formula was as follows:

$$Risk\;score=\sum_{i=1}^{n}XARGi*CoefARGi$$

Where *XARGi* represents the expression of each anoikis-related gene and the corresponding *CoefARGi* represents the regression coefficient. The median risk score threshold was used to distinguish the high-risk group from the low-risk group. The time-dependent ROC curves and the area under the curve (AUC) were established to evaluate the risk model. Multivariate Cox regression analyses were used to assess the correlation between clinical features and prognosis, and to determine whether the features could be independent prognostic factors. A nomogram was generated to predict the survival of patients based on risk scores through the summation of each prognostic clinical variable, and calibration curves were constructed to assess the accuracy of the risk model.

### Immune cell infiltrates and immune microenvironment

The relationship between risk groups and 22 distinct leukocyte subsets were identified using CIBERSORT algorithm. ESTIMATE algorithm was carried out to calculate tumor microenvironment scores and research the difference of immune and stromal scores between the high- and low-risk groups.

### Analysis of sensitivity to clinical therapies

OncoPredict, an R package for predicting drug responses and biomarkers (Maeser et al., 2021), was used to analyze the sensitivity of common oncological therapeutic drugs in TCGA-LUAD dataset. Drug susceptibility documentation and risk files were subsequently processed to draw boxplots of the two risk groups.

### Statistical analysis

The annotation and collation of transcriptome data, clinical and gene expression data were performed using PERL (version 5.32.0.1). Other statistical procedures were performed with R software (Version 4.1.2; Version 4.2.1). The Wilcoxon test was used to analyze anoikis-related variation according to sample quantity.

## Results

### Acquisition of differential ARGs and CNV analysis in LUAD

**FIGURE 1:**
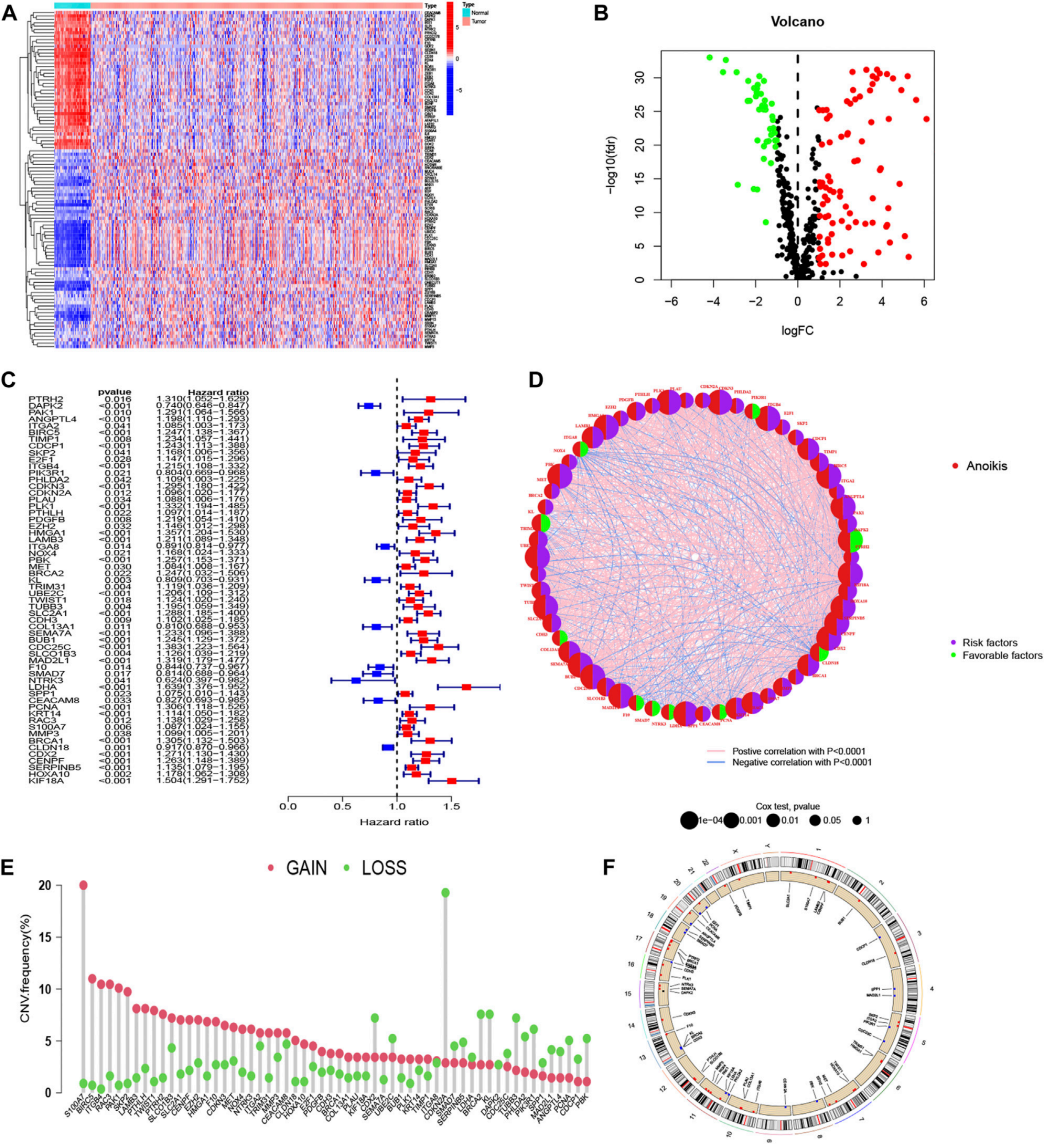
Acquisition of ARGs and CNV analysis. **(A, B)** Different expression of ARGs in LUAD with established standards (|log2FC| > 1 and FDR <0.05). **(C, D)** Forest plot and network graph of identified 58 ARGs correlated with prognosis *via* univariate Cox regression analysis. The significance criteria were set as *p*-value < 0.05. **(E)** The CNV frequency of gaining or losing in each ARGs. **(F)** Location and copy number of ARGs on chromosomes presented by CNV cycle diagram. Red spots mean gain, accordingly, blue one means loss.

To identify ARGs in LUAD, we abstracted 598 samples from the TCGA-LUAD cohort (59 normal and 539 tumor tissues) and 183 adenocarcinomas tissues from the GSE41271 dataset. A total of 640 ARGs were identified from GeneCards and Harmonizome platform. We utilized “limma” package to locate ARGs with significant variation in TCGA cohort, and the differential expression levels were presented in heatmap and volcano map (Figure 1A, B). The heatmap showed the top 50 significant ARGs in upregulation and downregulation. The red dots and green dots represented upregulated and downregulated genes in volcano map, respectively. The detail results of differential ARGs are attached in Supplementary Table S1. After merging gene expression information from TCGA cohort and GSE41271 dataset, we abstracted the expression of 111 differential ARGs (Supplementary Table S2) and combined it with survival materials to perform survival analysis. 58 ARGs were eventually identified from the 111 ARGs using univariate cox regression analysis and were illustrated in the forest plot and corresponding network visualization. 10 of 58 ARGs, such as PIK3R1 and ITGA8, were marked in blue with an HR < 1 and meant favorable factors of prognosis. Whereas the rest genes were identified with an HR > 1 and represented poor prognosis (Figure 1C). The correlation between these ARGs and prognosis were more directly exhibited in Figure 1D. The CNV frequency of 58 ARGs that had corresponding CNV data is shown in Figure 1E. As illustrated in Figure 1E, the frequency of gain of CNV on the left was obviously higher than the loss. Chromosome 7 and chromosome 11 both had four genes displayed as a “gain” in the copy number cycle diagram shown in Figure 1F.

### Two ARGs patterns identified by 58 significant ARGs

**FIGURE 2:**
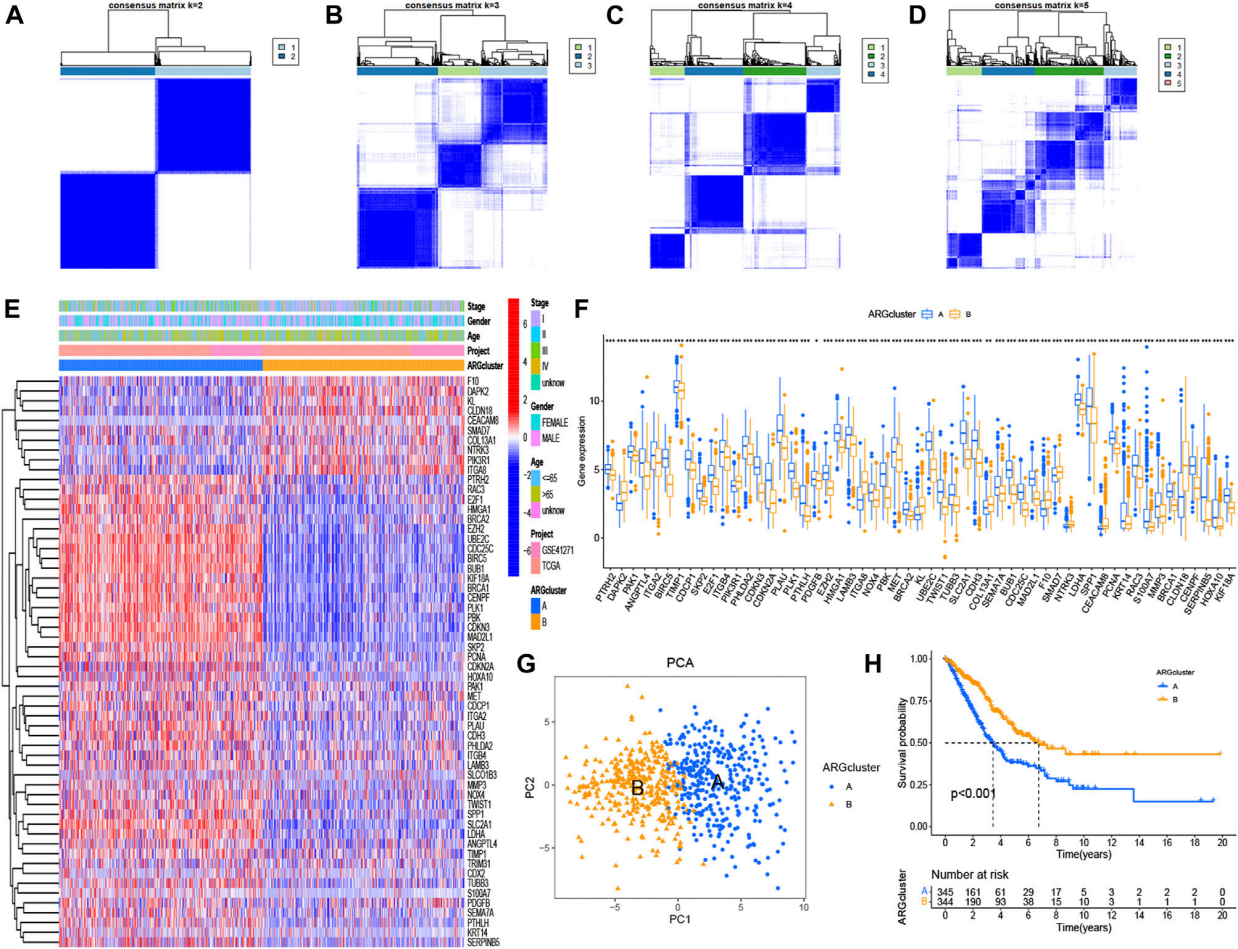
Consensus clustering of ARGs and survival analysis using K-M curves. **(A–D)** Consensus matrices of 58 significant ARGs (k = 2–5). **(E, F)** Heatmap and histogram of the 58 ARGs expression. **(G)** PCA for the expression profiles of different patterns. **(H)** Comparison of survival possibility in two subgroups. The significance criteria were set as *p*-value < 0.05. *p < 0.05, **p < 0.01, and ***p < 0.001.

When divided into two groups, the 58 significant ARGs had a significant difference using consensus clustering method (Figures 2A–D). Figures 2E, F based on cluster A and cluster B demonstrated the differential expression levels of these 58 significant ARGs. As the histogram shown, 55 ARGs had remarkable difference of expression in the two clusters, except for CDX2, SLCO1B3, TRIM31. Most of the expression of these genes were obviously higher in cluster A than in cluster B. Such as PHLDA2, CDKN3, CDKN2A, PLAU, PLK1 and so on. A PCA graph was drew for the verification of cluster allocation based on the ARGs (Figure 5G). It turned out that ARGs could classify the two patterns. Figure 5H showed that survival (*p* < 0.001) differed significantly between the two cluster subgroups, with a better survival rate in the cluster B than in the cluster A.

### Identification of KEGG pathway and immune analysis

**FIGURE 3:**
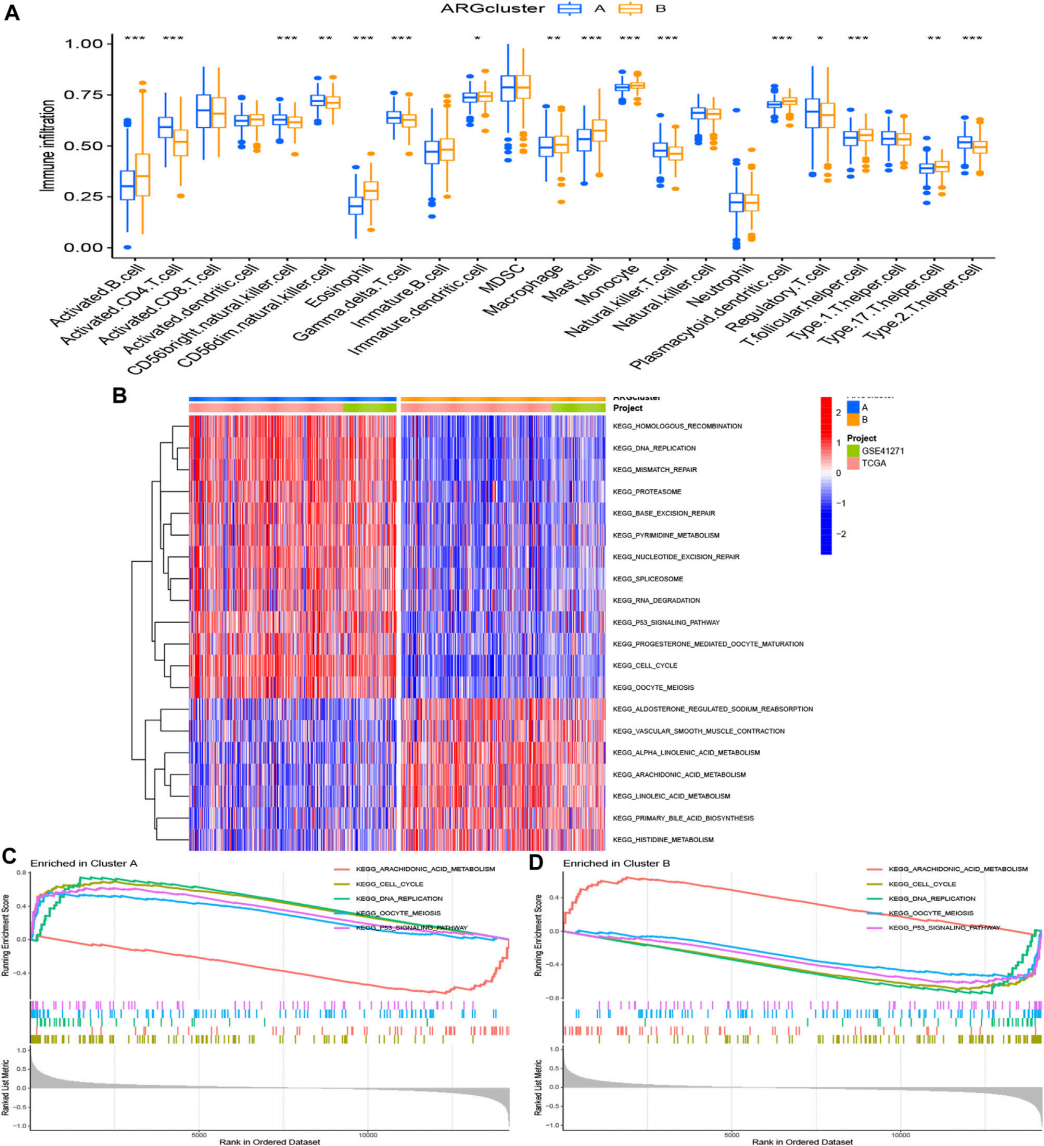
Gene set enrichment analysis. **(A)** Expression differences of immune cell infiltration between ARG cluster A and **(B)** *p < 0.05, **p < 0.01, and ***p < 0.001. **(B–D)** The KEGG enrichment analysis for the anoikis-related differentially expressed genes uncovered potential mechanism on the occurrence and development of the two patterns. Construction of cuproptosis-related risk model. **(A)** The point of minimum error was picked out by vertical dotted line based on LASSO cross validation. **(B)** Dynamic process diagram of Lasso filtering variables.

Immune-related analyses were performed to compare the differences between two ARG clusters. We investigated the immune cell abundance landscape to explore the differences between subgroups using the ssGSEA algorithm. There were marked differences in activated B cell, activated CD4 T cells, CD56 bright natural killer cell, CD56 dim natural killer cell, eosinophil, gamma delta T cell, immature dendritic cell, macrophage, mast cell, monocyte, natural killer T cell, plasmacytoid dendritic cell, regulatory T cell, T follicular helper cell, type 17 T helper cell, type 2 T helper cell (Figure 3A).

KEGG analysis was applied for comparison of the KEGG pathways in two ARG clusters. 20 significant KEGG pathways were plotted in the heatmap (Figure 3B). 13 pathways were activated in ARG cluster A, and cluster B was on the contrary. The diagram in Figures 3C, D demonstrated that ARG cluster A and B were primarily enriched in arachidonic acid metabolism, cell cycle, DNA replication, oocyte meiosis and P53 signaling pathway. Only arachidonic acid metabolism pathway turned off (“ silenced”) in cluster A, while only it turned on (“ activated”) in cluster B.

### Establishment and assessment of an anoikis-related risk model

**FIGURE 4:**
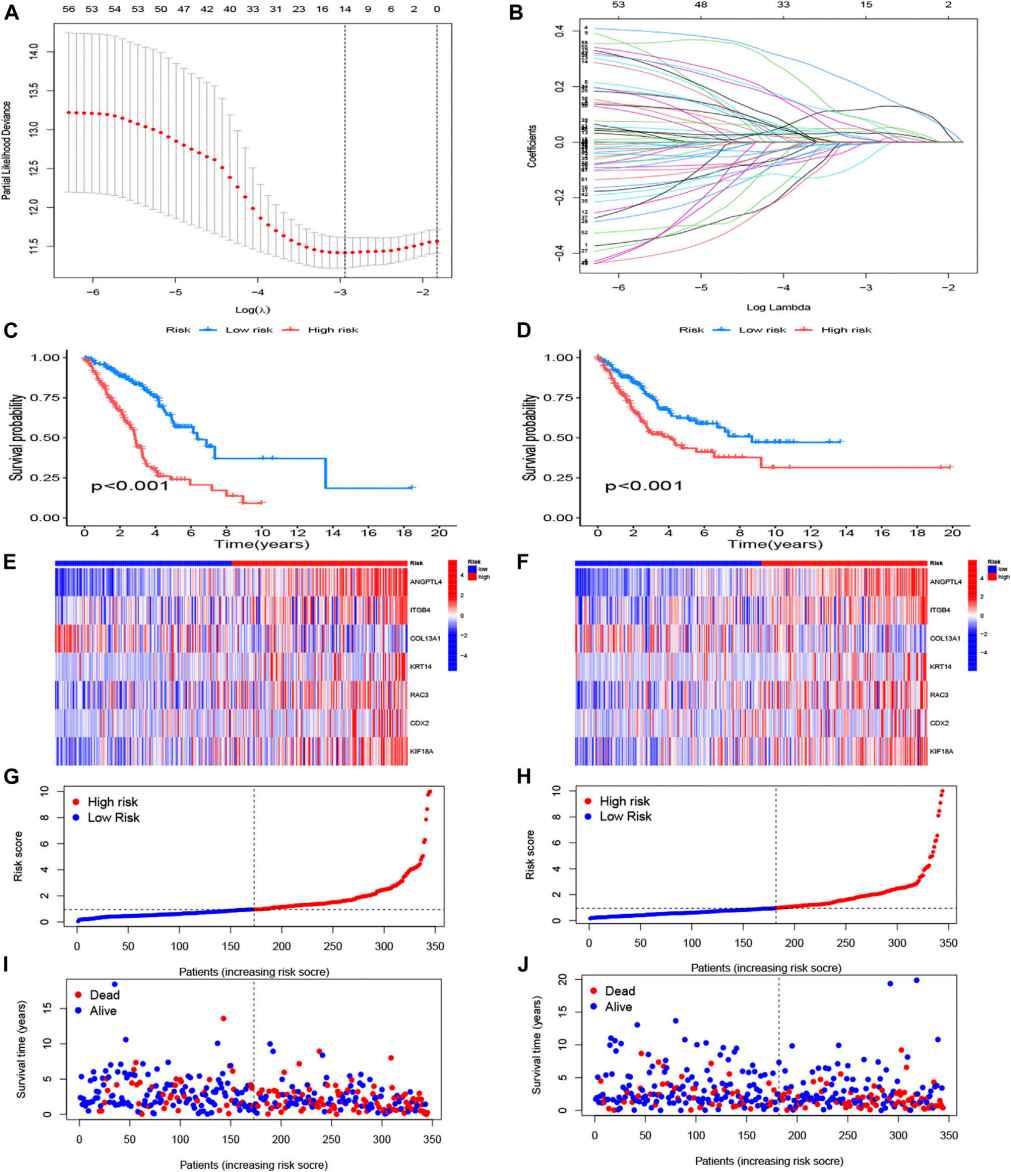
Construction of anoikis-related risk model. **(A)** The point of minimum error was picked out by vertical dotted line based on LASSO cross validation. **(B)** Dynamic process diagram of Lasso filtering variables. **(C, E, G, I)** Survival analysis and exposure rating of the training cohort. K-M survival analysis in training cohort **(C)**. Risk heatmap of training cohort indicated that six over-expressed ARGs in high-risk group, whereas the others over expressed in low-risk group **(E)**. Training cohort risk curve distinguished subgroups according to the median values **(G)**. Survival scatter diagram reflected a relationship between the risk score and survival time **(I)**. **(D, F, H, J)** Survival analysis and exposure rating of the testing cohort, utilizing K-M survival curve **(D)**, risk heatmap **(F)**, risk curve **(H)**, survival scatter diagram **(J)**. The significance criteria were set as *p*-value < 0.05.

The 58 ARGs were subjected to LASSO regression analysis and proportional-hazards model analysis with evaluation of prognosis. We identified the ARGs for the construction of the model after eliminating the effect of multicollinearity among the explanatory variables and model overfitting (Figures 4A, B). Ultimately, seven genes, consisted of angiopoietin-like 4 (ANGPTL4), integrin subunit beta 4 (ITGB4), collagen type XIII alpha 1 chain (COL13A1), KRT14, Rac Family Small GTPase 3 (RAC3), Caudal Type Homeobox 2 (CDX2), Kinesin Family Member 18A (KIF18A) were included to establish the prognostic risk model of LUAD. The risk scores of the seven screened ARGs determined by multivariate analysis are shown in Table 1. The risk score was calculated with the following formula:

$$\begin{array}{l} Risk\;score=\left(XANGPTL4\times 0.33420\right)+\left(XITGB4\times 0.13667\right)\\ -\left(XCOL13A1\times 0.33534\right)+\left(XKRT14\times 0.07749\right)+(XRAC3\\ \times 0.14982)+\left(XCDX2\times 0.18313\right)+\left(XKIF18A\times 0.22797\right) \end{array}$$

**TABLE 1:** Seven ARGs selected by multivariate cox results.

ARGs	Coef
ANGPTL4	0.33420
ITGB4	0.13667
COL13A1	−0.33534
KRT14	0.07749
RAC3	0.14982
CDX2	0.18313
KIF18A	0.22797

ARGs, anoikis-related genes; Coef, coefficient.

The risk model divided patients into two cohorts, a training group (n = 345) and a testing group (n = 344). Follow-up analyses were performed after comparing the clinical traits of the two cohorts without significant difference (Table 2). We then constructed the K-M survival curves to compare survival between high- and low-risk subgroups in the training and testing cohorts. There were marked differences between the risk subgroups, with lower survival rates in the high-risk group and higher survival rates in the low-risk group (Figures 4C, D). The risk scores were calculated and the median threshold of the risk score was set to 1 to distinguish between high- and low-risk groups (Figures 4G, H). Survival scatter diagrams of the two cohorts showed an inverse correlation between survival time and the risk score, indicating that patients in the high-risk group had a worse prognosis (Figure 4I, J). The risk heatmap of the training cohort indicated that six ARGs (ANGPTL4, ITGB4, KRT14, RAC3, CDX2, KIF18A) were overexpressed in the high-risk group, whereas COL13A1 was highly expressed in the low-risk group (Figure 4E). The testing cohort confirmed the expression of the six ARGs in high- and low-risk subgroups (Figure 4F).

**TABLE 2:** Clinical characteristics analysis of subgroups.

	(n, %)			
Covariates	Total quantity	Training	Testing	*p*-value
	(n = 689)	group	group	
		(n = 345)	(n = 344)	
Age				0.1108
≤65	317(46.01%)	171(49.57%)	146(42.44%)	
>65	362(52.54%)	172(49.86%)	190(55.23%)	
NA	10(1.45%)	2(0.58%)	8(2.33%)	
Gender				0.4242
Female	362(52.54%)	187(54.2%)	175(50.87%)	
Male	327(47.46%)	158(45.8%)	169(49.13%)	
Stage				0.1094
I	372(53.99%)	188(54.49%)	184(53.49%)	
II	148(21.48%)	62(17.97%)	86(25%)	
III	131(19.01%)	71(20.58%)	60(17.44%)	
IV	30(4.35%)	18(5.22%)	12(3.49%)	
NA	8(1.16%)	6(1.74%)	2(0.58%)	

NA, unknown variables; n, numbers of patients.

**FIGURE 5:**
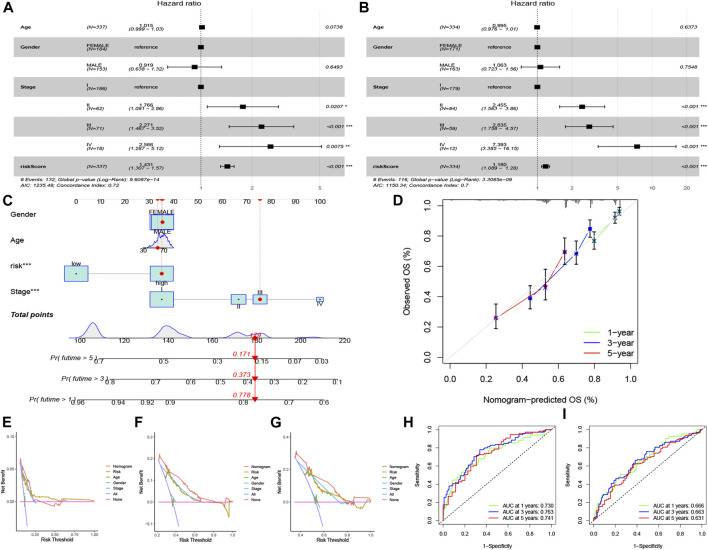
Independent prognostic analysis and nomogram construction for LUAD. Univariate Cox regression analysis in training group **(A)** and testing group **(B)** of clinical features in the risk model set (*p* < 0.05). **(C)** The construction of nomogram to foretell patient’s 1 -, 3 -, and 5-year survival rates, according to the total points. **(D)** Calibration curve of the nomogram. **(E–G)** C-index curve for different variables, including risk score, age, gender, and stage. ROC curves for 1 -, 3 -, and 5-year overall survival in training group **(H)** and testing group **(I)**.

The risk score and partial clinical traits were included in an independent prognostic analysis using univariate Cox regression. Both risk scores (*p* < 0.001) in training and testing group were associated with prognosis, suggesting that the risk score could be used as an independent predictor (Figures 5A, B). As shown in Figures 5C, D nomogram and calibration curve were established to predict the survival time of patients with LUAD. The C-index curve confirmed that the risk model constructed had the highest accuracy to predict survival compared with the clinical characteristics (age, gender, and stage) (Figures 5E–G). The ROC curve showed the highest AUC value of 0.763 for the risk score, and the AUC values for 1 -, 3 -, and 5-year OS were 0.730, 0.763, and 0.741, respectively (Figure 5H). The AUC values for 1 -, 3 -, and 5-year OS were 0.666, 0.663, and 0.631 in testing group (Figure 5I).

### Immune-related and drug sensitivity analyses based on the risk model

**FIGURE 6:**
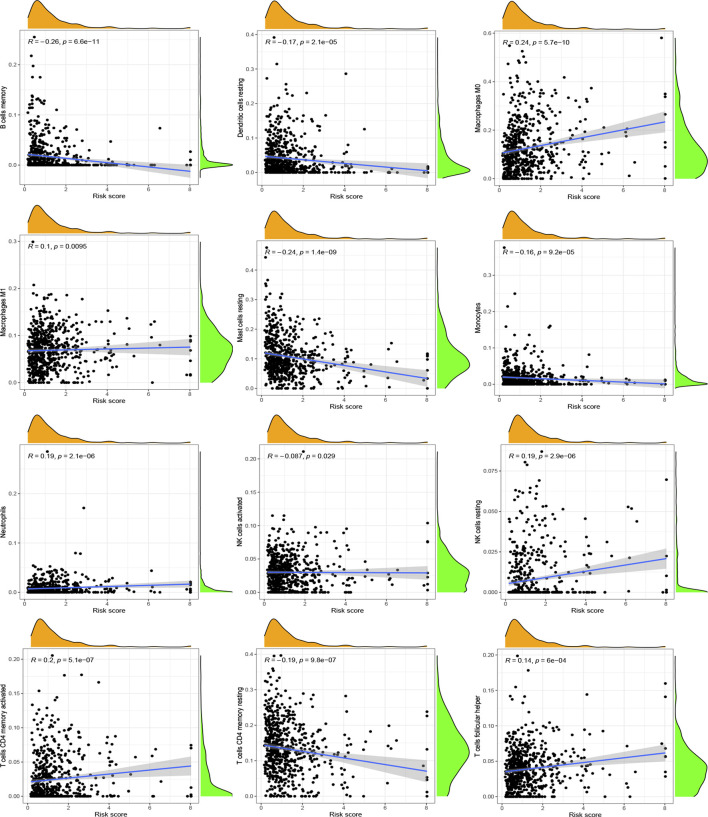
Correlation between immune cells and risk scores. Positive correlations: activated memory CD4 T cells, M0 macrophages, M1 macrophages, neutrophils, resting NK cells and follicular helper T cells. Negative correlations: activated mast cells, activated NK cells, memory B cells, monocytes, resting dendritic cells, resting mast cells and resting memory CD4 T cells. *p* < 0.05 was considered as significant correlation.

**FIGURE 7:**
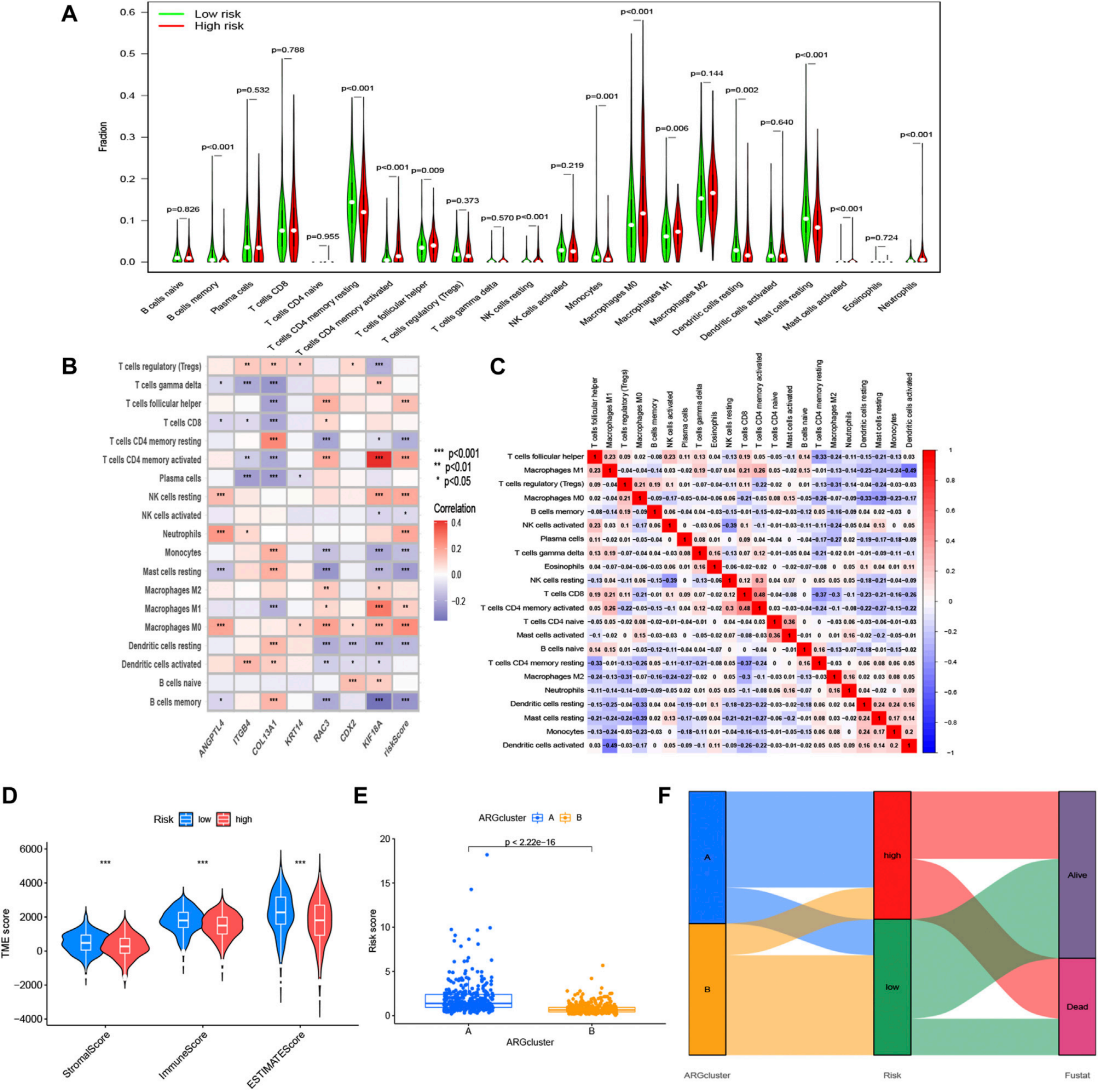
Immune-related analysis based on the risk model. **(A)** Expression differences of immune cell infiltration in risk subgroups. **(B)** Correlation between 7 ARGs, risk scores, and the immune cells. **(C)** Correlation between these immune cells. **(D)** Other characteristics including estimate score, immune score, and stromal score between the high- and low-risk groups. **(E)** Differential risk score comparison in the two ARGs patterns. **(F)** Sankey diagram of the relationship between two ARGs patterns, survival status, and risk scores. *p < 0.05, **p < 0.01 and ***p < 0.001.

After investigating the immune cell abundance using the CIBERSORT algorithm, we compared the correlation between immune cell content and risk scores. The correlation analysis plot demonstrated that 12 immune cells had significant associations with risk scores (Figure 6). There were remarkably positive correlations in activated memory CD4 T cells, M0 macrophages, M1 macrophages, neutrophils, resting NK cells and follicular helper T cells. The others, including activated mast cells, activated NK cells, memory B cells, monocytes, resting dendritic cells, resting mast cells and resting memory CD4 T cells, had negative correlations with risk scores. In addition, we analyzed immune cells expression in the two risk groups and identified 11 significantly different immune cells (Figure 7A). A heatmap of the correlation of immune cells with the seven model ARGs was generated and presented a close connection between KIF18A and the cells (Figure 7B). The correlations between these 22 immune cells which were allocated for immune analyses were found in Figure 7C. Activated memory CD4 T cells showed high positive correlations with CD8 T cells. The low-risk group got higher immune score (*p* < 0.001) and stromal score (*p* < 0.001) than the high-risk group (Figure 7D).

Considering the relevance of ARGs clusters to risk model, we further scored the two clusters and compared the difference of the risk score, and the risk scores in A group was higher than that in B group (Figure 7E). The sankey diagram distinctly displayed the association between clusters and anoikis-related risk scores (Figure 7F).

**FIGURE 8:**
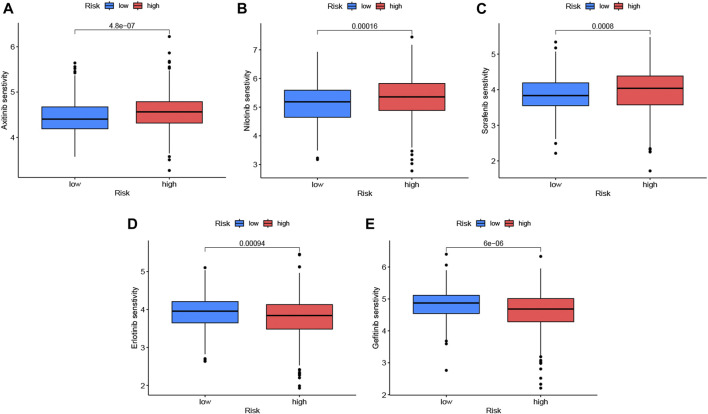
Drug sensitivity analysis in low-risk and high-risk sets. We listed 5 kinds of anticancer drugs, including axitinib **(A)**, nibtinib **(B)**, sorafenib **(C)**, erlotinib **(D)** and gefitinib **(E)**, with blue boxplots represented low-risk group and red expressed high-risk group. Erlotinib and gefitinib which were common targeted drugs to LUAD were not a better choice for patients with high-risk scores. The significance criteria were set as *p*-value < 0.05.

The correlation analysis of IC50 with risk scores showed that 73 anticancer drugs had significant correlations with risk scores, including 17 drugs with negative associations and 56 drugs with positive associations. This indicated that patients in the high-risk group responded well to most medications. Detailed information is found in Supplementary Table S3. The 3 kinds of molecular targeted drugs with significant differences (*p* < 0.05) are shown in Figures 8A–C, which might be candidate medications for patients with LUAD in the high-risk group. In addition, there were 2 types of frequent medications (erlotinib and gefitinib) associated with LUAD and had lower sensitivity in high-risk group (Figures 8D, E).

### Analysis of ARGs based on single-cell RNA sequencing data

**FIGURE 9:**
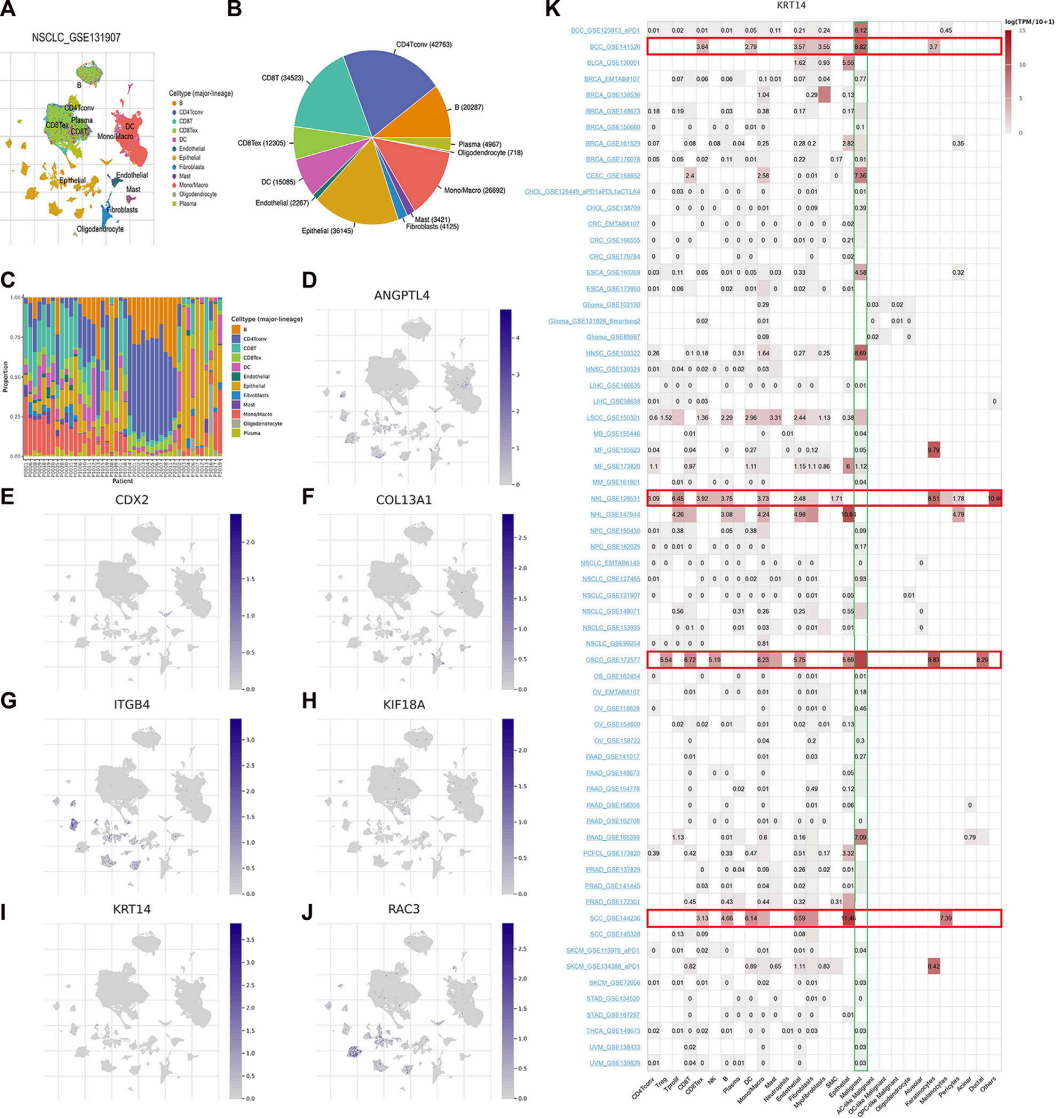
Seven ARGs analyses based on single-cell RNA sequencing data. **(A)** Annotation of cell types in GSE131907. Proportion of each cell type in the dataset **(B)** and in each patient **(C)**. **(D–J)** Expression of ANGPTL4, CDX2, COL13A1, ITGB4, KIF18A, KRT14, and RAC3. **(K)** Expression of KRT14 in pan-cancer.

GSE131907, a single-cell RNA sequencing data of LUAD, was chosen to exhibit the expression of seven model ARGs utilizing TISCH2 platform. The landscape of intermediate cells was shown in Figure 9A. The pie diagram depicted the proportion of each cell type (Figure 9B). As it illustrated, CD4 conventional T cell was the most abundant immune cell. Following the research of proportion of each cell type in patients (Figure 9C), the expression of seven ARGs was analyzed and draw in Figure 9D–J. ANGPTL4, CDX2, ITGB4, KRT14, and RAC3 were mainly enriched in epithelial cells. COL13A1 was mainly expressed in fibroblasts and KIF18A was mainly expressed in CD8 T cells. Subsequently, we explored expression of these ARGs in pan-cancer single-cell sequencing dataset and found that only KRT14 was highly expressed in various tumors, including basal cell carcinoma (BCC), non-hodgkin lymphoma (NHL), oral squamous cell carcinoma (OSCC) and squamous cell carcinoma (SCC). In various tumors, KRT14 was widely expressed in malignant cells in the tumor microenvironment (Figure 9K).

## Discussion

Despite advances in therapeutic modalities for malignant lung tumors, sundry drugs can result in a poor prognosis because of undesirable side effects, resistance, and cachexia after progression (Wang et al., 2021). Chemoresistance, particularly, remains an important cause of failure of treatment and the driving force of death. Inherent resistance leading to poor initial treatment response and acquired resistance leading to poor follow-up responses constitute the two aspects of resistance (Gottesman, 2002; Nikolaou et al., 2018; Jing et al., 2020). Chemotherapeutic drugs act on cancers by inducing programmed cell death, whereas tumor death evasion can increase the occurrence of resistance (Wattanathamsan et al., 2019; Yang et al., 2021). Several studies have focused on examining cell death mechanisms to overcome drug resistance. A previous review reported that dysregulation of ULK1-mediated autophagy plays a crucial role in drug resistance (Liu et al., 2020). Ferroptosis induces tumor cell death through lipid peroxidation mechanistically and *via* shrunken cell mitochondria morphologically (Dixon et al., 2012).

Our research focused on anoikis in LUAD, which is one of the programmed cell death. We hypothesized that ARGs act on lung tumor cells in a similar way as ferroptosis-related or cuproptosis-related genes identified in previous research (Tian et al., 2021; Zhang et al., 2022). 58 ARGs were identified for the establishment of anoikis-related risk model and the ARGs clustering analysis. The feasibility of the model based on seven ARGs was verified using a variety of analytical methods. And it implied that ANGPTL4, CDX2, ITGB4, KRT14, RAC3, and KIF18A were potential therapeutic targets for LUAD. Regarding drug resistance, the anoikis-related risk model analyzed hundreds of anticancer medicines to compare drug responses in the risk subgroups and provide treatment strategies for LUAD. Both training ang testing groups demonstrated that patients prognosis could be accurately predicted by anoikis-related model. Subsequently, the correlations between the immune cells and risk scores, risk subgroups and seven ARGs were taken into consideration. Patients with increasing M0 macrophages were linked with graver prognosis (Liu et al., 2017; Yi et al., 2021), and the linear relation between risk scores and M0 macrophages in our research also consisted with the previous study. Conversely, memory B cells had a negative relation with risk scores. It was reported that memory B cells was the foundation for having lasting immunity (Fan et al., 2021) and it was closely associated to superior survival (Zhang et al., 2019). We also found immune score and stromal score were both higher in low-risk group, implying that TME scores may exhibit a distinct prognostic value in LUAD (Wu et al., 2021).

We analyzed immune microenvironment, KEGG analysis and prognosis to explore two distinct ARGs patterns. Patients with poor prognosis in cluster A were notably more than cluster B. In addition, increased number of activated CD4 T cells, CD56 bright natural killer cell, CD56 dim natural killer cell, gamma delta T cell, natural killer T cell, regulatory T cell, type 2 T helper cell in cluster A was associated with poor prognosis in LUAD. KEGG analyses showed that genes were mainly involved in arachidonic acid metabolism, cell cycle, DNA replication, oocyte meiosis and P53 signaling pathway, confirming differences in immune factor abundance and immune activity between the two groups. Moreover, a high inhibition of arachidonic acid metabolism and activation of cell cycle, DNA replication, oocyte meiosis and P53 signaling pathway may lead to poor prognosis in LUAD.

We performed the single-cell analysis to seek the cell types that express the model ARGs. The expression level of KRT14 in various cell types, including immune cells, malignant cells, stromal cells, and functional cells, was explored. It depicted that KRT14 was mainly overexpressed in malignant cells and immune cells (especially epithelial cells, CD8 T cells, endothelial cells, monocyte/macrophage). Several studies have reported that high KRT14 expression can cause poor prognosis in cancers. KRT14 associated with TRAIL and TNF receptor signaling pathway led to a worse prognosis in LUSC (Dong et al., 2020) and KRT14 following the upregulation of Gkn1 could inhibit anoikis and contribute to lung nodal metastasis and poor survival (Yao et al., 2019). In breast cancer, KRT14^+^ epithelial tumor cell clusters promoted to distant organs metastases (Cheung et al., 2016). The underlying correlation between KRT14 and pan-cancer needs to be analyzed further.

The present results are theoretical results based on bioinformatics. Because this is a novel topic, information about the ARGs identified, including CNV annotation, mechanistic studies, and confirmation *via in vivo* and *in vitro* experiments require further study. Cell line experiments, preclinical research, and clinical trials are needed to further detect the expression and mechanism of these ARGs.

## Conclusion

This study combines bioinformatics research with ARGs to construct a risk model as a prognostic index for application in clinical practice. The feasibility of the anoikis-related model will be validated through additional clinical trials. We expect that further literature will be published to uncover the essential mechanisms underlying the function of ARGs in tumor cells and to identify new anoikis-related tumor therapies.

## Data Availability

The datasets presented in this study can be found in online repositories. The names of the repository/repositories and accession number(s) can be found in the article/Supplementary Material.
